# The effect and mechanism of miR-30e-5p targeting SNAI1 to regulate epithelial-mesenchymal transition on pancreatic cancer

**DOI:** 10.1080/21655979.2022.2050880

**Published:** 2022-03-18

**Authors:** Ziyu Liang, Shaomei Tang, Rongquan He, Wei Luo, Shanyu Qin, Haixing Jiang

**Affiliations:** a Department of Gastroenterology, The First Affiliated Hospital of Guangxi Medical University, Nanning, Guangxi, China; b Department of Oncology, The First Affiliated Hospital of Guangxi Medical University, Nanning, Guangxi, China

**Keywords:** miR-30e-5p, SNAI1, EMT, pancreatic cancer

## Abstract

Accumulating evidence indicates that abnormally expressed microRNAs (miRNAs, miRs) contribute to cancer progression. Nonetheless, the role of miR-30e-5p in pancreatic cancer (PCa) remains unclear. In this study, using quantitative real-time polymerase chain reaction analysis, we found that miR-30e-5p expression was downregulated in human PCa tissues compared with that in normal para-cancerous tissues. After transfecting with miR-30e-5p inhibitors, miR-30e-5p mimics, or empty vectors in the BxPC-3 and PANC-1 cells, respectively, the experiments revealed that the upregulation of miR-30e-5p expression inhibited cell growth, invasion, migration and epithelial-mesenchymal transition (EMT), and promoted apoptosis, while miR-30e-5p downregulation had the opposite effects. RNA sequencing of miR-30e-5p inhibitor-, miR-30e-5p mimic-, and the negative control (NC)-treated groups revealed that miR-30e-5p may affect epithelial cell differentiation, cell growth and death. Next, the snail family transcriptional repressor 1 (SNAI1) was predicted and verified as the target gene of miR-30e-5p using bioinformatics analysis and luciferase assays. SNAI1 expression levels were decreased in the PCa cells transfected with miR-30e-5p mimics, whereas the opposite was observed in the cells transfected with miR-30e-5p inhibitors. Subsequently, PCa cells were transfected with a vector overexpressing SNAI1 (OE-SNAI1) and miR-30e-5p mimics, miR-30e-5p inhibitors, or empty vectors. Compared with that in the OE-SNAI1 + miR-30e-5p NC group, transfection with OE-SNAI1 + miR-30e-5p mimics inhibited the PCa cell growth, migration, and increased apoptosis, whereas transfection with OE-SNAI1 + miR-30e-5p inhibitors had the opposite effects. In conclusion, miR-30e-5p potentially inhibits PCa cell proliferation, migration, and invasion via the SNAI1/EMT axis.

## Introduction

Pancreatic cancer (PCa) is a challenging disease worldwide owing to the difficulties in its early diagnosis along with its high malignancy, rapid progression, and poor curative effect [1,2]. Its incidence is increasing annually, and it is projected to become the second leading cause of cancer-related mortality in the United States over the next 30 years [3]. Currently, the best curative therapy for PCa is surgery, which is only suitable for 10–15% of patients; 80–85% of patients appear to have either unresectable or metastatic PCa [4]. Due to the limitations of early molecular detection methods, effective molecular biomarkers for the screening, diagnosis, and prognostic evaluation of PCa have not yet been identified. Therefore, there is an urgent need to gain insights into the molecular mechanisms of PCa to identify novel targets for the development of precise and effective treatment strategies.

With the development of high-throughput sequencing technology, non-coding RNAs have attracted increasing attention in recent years, as they have greatly enriched the molecular map of malignant tumors [5,6]. MicroRNAs (miRNAs, miRs) are small noncoding RNAs with a length of 18–25 nucleotides. They act mainly on the 3’-untranslated region (3’-UTR) of target genes in a complementary or reverse complementary manner, thus regulating the gene expression [7]. Accumulating evidence indicates that miRNAs are abnormally expressed in cancers and may act as tumor suppressors or oncogenes by affecting the growth, development, invasion, and distant metastasis of cancers [8,9]. Among these miRNAs, miR-30e functions as a tumor suppressor in various human cancers, including liver cancer [10], colorectal cancer [11], and prostatic cancer [12]. In contrast, miR-30e promotes tumor invasion and metastasis as an oncogene in malignant salivary gland tumors [13] and lung adenocarcinoma [13]. These results suggest that miR-30e is a potential therapeutic agent for cancer treatment. Nevertheless, the roles and mechanisms of miR-30e in PCa remain unclear.

EMT is a process in which epithelial cells lose tight junction characteristics and polarity and transform into mesenchymal cells with the ability to migrate under certain conditions [14]. E-cadherin, a hallmark of EMT, is often downregulated during cancer occurrence and development. Additionally, upregulations of N-cadherin, Snail family transcriptional repressor 1 (SNAI1), and matrix metallopeptidase 9 (MMP-9) expression are characteristics of EMT [15]. EMT is vital for the occurrence and metastasis of PCa, and numerous miRNAs are involved in this process [16–18]. Recently, Xue et al. demonstrated that inhibiting miRNA-539 advances the proliferation, migration, and EMT of PCa cells by targeting the SP1 translocation factor [19]. Another study reported that miR-145 may suppress EMT in PCa cells by inhibiting the transforming growth factor-β signaling pathway [20]. A nearby research discovered that miR-490-5p exhibits anti-cancer role in PCa through targeting MAGI2-AS3 and regulating EMT [21]. However, whether miR-30e-5p plays a significant role in the carcinogenesis of PCa via EMT remains unclear. To date, only few studies have investigated the functions and molecular mechanisms of miR-30-5p in the onset and progression of PCa.

It is hypothesized that miR-30e-5p may restrain cell proliferation, migration, invasion and advance cell apoptosis in the PCa via the SNAI-mediated EMT pathway. Thus, the aim of this study was to identify the roles of miR-30e-5p in cell proliferation, invasion, migration, apoptosis, and EMT in PCa clinical tissues and cell lines, and further explore its target genes and potential mechanisms during carcinogenesis and development of PCa. These experimental results will aid in the development of targeted therapeutic strategies for PCa.

## Materials and methods

### Patient tissue specimens

A total of 48 paired human PCa and adjacent non-tumor tissues were obtained from the First Affiliated Hospital of Guangxi Medical University between July 2016 and August 2019. All patients were verified as primary pancreatic carcinomas by two professional pathologists and did not perform chemotherapy, radiotherapy or immunotherapy before resection. All patients had signed written informed consent, and this study was approved by the ethics committee of the First Affiliated Hospital of Guangxi Medical University.

### Cell culture and transfection

The human PCa cell lines (BxPC-3 and PANC-1) were purchased from Shanghai Institute of Biochemistry and Cell Biology (Shanghai, China), and cultured in the Dulbecco’s modified eagle medium (DMEM) (Gibco, USA) with 1% penicillin/streptomycin (Guangxi, China) and 10% fetal bovine serum (FBS; Gibco, USA). Cells were kept at 37°C in an incubator with containing 5% CO_2._ Lipofectamine 2000 was applied to implement cell transfection in line with the protocol of the manufacturer. miR-30e-5p mimics, inhibitors, or empty vector were transfected into BxPC-3 and PANC-1 cells, respectively. Also, SNAI1 overexpression lentivirus was co-transferred into PCa cell lines [22].

### Quantitative reverse‐transcription polymerase chain reaction (qRT-PCR)

The analysis of relative gene expression data was based on the previously reported method [23]. Total RNA was extracted from tissues and cells utilizing miScript PCR System (QIAGEN, Germany) on the basis of operator’s instructions. Amplified reaction was performed in the ABI7500 system (Applied Biosystems, USA). Primers for miR-30e-5p and internal control U6 were as following: miR-30e-5p: forward primer: 5’-CGGGCTGTAAACATCCTTGAC-3’; reverse primer: 5’-GTCGTATCCAGTGCAGGGTCCGAGGTATTCGCACTGGATACGACCTTCCA-3’; U6: forward primer: 5’-CTCGCTTCGGCAGCACA-3’; reverse primer: 5’-AACGCTTCACGAATTTGCGT-3’. Primers for SNAI1 and internal control GAPDH: SNAI1: forward primer: 5’- GAGCCCAGGCAGCTATTTCA-3’; reverse: 5’- CATCGGTCAGACCAGAGCAC-3’; GAPDH: forward 5’- CCAGGTGGTCTCCTCTGA-3’; reverse: 5’- GCTGTAGCCAAATCGTTGT-3’. The relative expressions of miR-30e-5p, normalized to U6, were calculated by using the 2− ΔΔCt method.

### Cell proliferation assay

Mechanism of cellular 3-(4,5-dimethylthiazol-2-yl)-2,5-diphenyltetrazolium bromide (MTT) Assay Kit was used to evaluate PCa cell proliferation. In a nutshell, using seeding transfected cells into 96-well plates with a density of 7000 cells per well; then adding 20 ul MTT solution after incubating 24 hours, 48 hours, 72 hours and 96 hours, respectively; culturing in 37°C incubator for 4 hours; adding 150ul dimethyl sulfoxide (DMSO) and oscillating 10 minutes; lastly examining optical density (OD) at 490 nm on a microplate reader. The formula of calculating cells using cell count plates: the cell numbers in the per milliliter cell suspension = total cell numbers in the four big squares/4 * 10,000. The cell survival fraction = Experimental groups (OD)/Control groups (OD) [22,24].

### Colony formation assay

Transfected PCa cells were inoculated into six-well plates with a density of 500 per well and placed in the incubator containing 5% CO2 at 37°C. After three weeks, fixing with paraformaldehyde for 15 minutes and staining crystal violet for 15 minutes. The clone number of more than 50 cells was counted and recorded under microscope [22].

### Cell apoptosis assay

The cell suspension was regulated as 1 * 10^6^ cells/ml using cell count plates. Then, PCa cells were gather and washed twice with Binding Buffer after a 48-hours transfection. Annexin V-FITC and Propidium Iodide were mixed into cells and incubated in the dark for 15 minutes. Finally, flow cytometry was used to detect cell apoptosis [25].

### Cell invasion assay

Transfected PCa cells were prepared into cell suspension according to 7000 cells per well. Afterward, 150 μl serum-free cell suspension was added into the upper chamber, while 600 μl complete medium with 10% FBS was added into the lower chamber. After incubating for 48 hours at 37°C, cells in the upper chamber were erased and other cells was stained with 0.1% crystal violet for 20 minutes and washed twice with phosphate buffer saline (PBS). Invasive cells were watched and photographed under microscope [22].

### Cell migration assay

Transfected PCa cells were cultured with a monolayer and scratched perpendicularly to plate bottom. Thereafter, washing with PBS three times, replacing the medium and incubating. The migrated distance was photographed and calculated at 0 and 48 hours after scratching [22].

### Luciferase assay

The SNAI1 3’-UTR with the miR-30e-5p binding site was insert into psiCheck2-reporter vector to construct SNAI1 mutated and wildtype vectors (SNAI-MUT and SNAI1-WT). By utilizing Lipofectamine 2000 (Invitrogen; Thermo Fisher Scientific, Inc.), SNAI-MUT or SNAI1-WT were co-transfected into 293 T cells with miR-30e-5p mimics following the manual. Luciferase activity was gauged 48 hours later with the dual-luciferase assay system (Promega) [22].

### Western blotting

The intracellular protein was extracted with protein lysate (Beijing Solarbio Science & Technology Co., Ltd.), and the protein concentration was measured employing Bicinchoninic Acid (BCA) assay kit (BOSTER). A total of 50 µg protein was segregated on 8‑10% SDS‑PAGE and diverted to polyvinylidene difluoride membranes (PVDF), then sealed with 5% skim milk at room temperature for 2 hours. Primary antibodies: SNAI1 (dilution,1:500; cat. no. ab82846; Abcam), E-cadherin (dilution, 1:1000; cat. no. ab15148; Abcam), N-cadherin (dilution, 1:1000; cat. no. ab18203; Abcam), MMP-9 (dilution, 1:500; cat. no. ab38898; Abcam) and β-actin (cat. no. ab8227; Abcam 1:5000). The primary antibody was incubated overnight in a refrigerator at 4°C. Next, the horseradish peroxidase labeled secondary antibody (dilution 1:5000; cat. No. ab6721; Abcam) was added and incubated at room temperature for another hour. Visualization of protein bands were obtained based on an Odyssey infrared laser imaging system. Lastly, using β-actin as internal reference, the protein relative expressions of SNAI1, E-cadherin, N-cadherin and MMP-9 were calculated using Image J [22].

### RNA sequencing

The analytical procedures of RNA sequencing are in line with the previously published articles [26,27]. PANC-1 cells from miR-30e-5p mimics, inhibitor, and NC groups were performed RNA sequencing with three biological repetitions. Total RNA was drawn from PANC-1 cells using Trizol reagent. According to manufacturer’s recommendations, the integrity and quantity of total RNA were assessed. Then, library construction and sequencing were carried out at the company GENE DENOVO in Guangzhou, China.

### Bioinformatics analysis

For the RNA sequencing data, following standard process [28], FastQC was used for quality control, human genome (hg19) was the reference genome for mapping to reads by HISAT2, and StringTie was used to quantify with Fragments Per Kilobase Million (FPKM). Differential expressed genes were defined as p < 0.05 and absolute foldchange>1.2 according to the results from DESeq2. Functional annotations were conducted using Metascape [29]. And volcano plots were drawn using R statistical software (version 5.2). For predicting the targeted genes of miR-30e-5p, starBase v3.0 was employed [30].

### Statistical analysis

SPSS version 23.0 was employed to conduct statistical analysis and pictures were drawn using GraphPad Prism 8.3. The Shapiro–Wilk method is used to test whether the measurement meets the normal distribution, and the Levene method is used to test whether the measurement obeys the homogeneity of variance. The results found that they are all approximately obey the normal distribution, and meet the homogeneity of variance. Therefore, the means of the two groups are compared by Student’s t-test, and the means of three groups and above are compared by one-way ANOVA. If the difference between groups is statistically significant, the Bonferroni test is further used for multiple comparisons to adjust the test level and clarify the specifics. The mean (M) and standard deviation (SD) from at least three independent repeats were used to present all data. The p < 0.05 was ascertained to be statistically significant. All experiments were repeated three times [31].

## Results

The goal of this study is probing the role of miR-30-5p and latent mechanisms in the occurrence and development of pancreatic cancer (PCa). Briefly, we found that up-regulating miR-30e-5p expression inhibited PCa cell growth, invasion, migration and epithelial-mesenchymal transition (EMT) and induced apoptosis, whereas lessening its expression had opposite effects. And Snail family transcriptional repressor 1 (SNAI1) was predicted and verified as a target gene of miR-30e-5p by utilizing bioinformatic analysis and luciferase assays. Subsequently, PCa cells were transfected with a vector overexpressing SNAI1 (OE-SNAI1) and miR-30e-5p mimics, miR-30e-5p inhibitor, or empty vector, and then cell proliferation, clone formation, apoptosis, invasion and migration assays were implemented. The results indicated that compared with the OE-SNAI1 + miR-30e-5p negative control (NC) group, transfection with OE-SNAI1 + miR-30e-5p mimics inhibited PCa cell growth, and migration and increased apoptosis, while similarly, OE-SNAI1 + miR-30e-5p inhibitor had the opposite effect.

### miR-30e-5p expression was descending in PCa tissues

**Figure 1.**
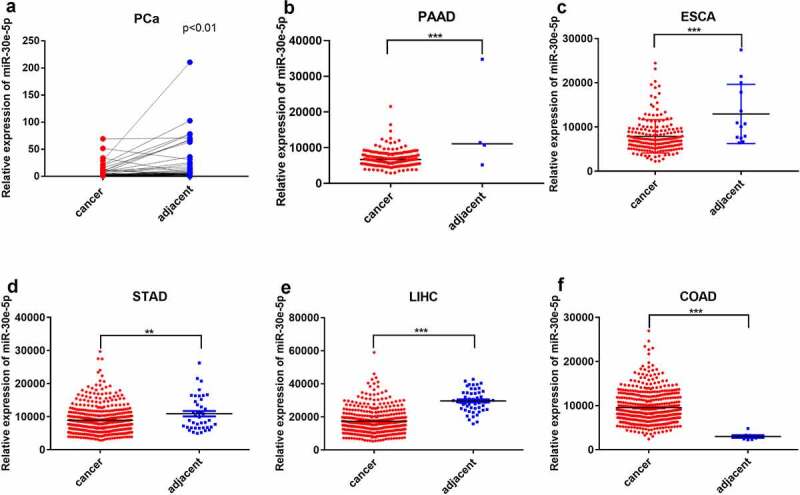
The expression of miR-30e-5p was descending not only in PCa tissues but also in other pan-gastrointestinal cancers tissues except for COAD, compared to normal tissues. (A) qRT-PCR analysis of miR-30e-5p expression in 48 paired PCa tissues (cancer) and adjacent non-cancer tissues (adjacent). (B-F) miR-30e-5p expression in PAAD, ESCA, STAD, LIHC, COAD cancer and adjacent normal tissues based on TCGA miRNA-seq. ***p < 0.001; **p < 0.01; *p < 0.05. Abbreviations: PCa: pancreatic cancer; PAAD: Pancreatic adenocarcinoma; ESCA: Esophageal carcinoma; STAD: Stomach adenocarcinoma; LIHC: Liver hepatocellular carcinoma; COAD: Colon adenocarcinoma.

First, we examined miR-30e-5p expression in 48 paired PCa and para-cancerous normal tissues using quantitative real-time (qRT)-PCR. miR-30e-5p expression was markedly lower in PCa tissues than in normal tissues (p < 0.05; Figure 1A). We further surveyed miR-30e-5p expression using the Cancer Genome Atlas database (TCGA). As expected, miR-30e-5p expression was significantly decreased in PCa tissues compared with that in adjacent normal tissues (p < 0.01; Figure 1B). Despite all this, it is interestingly that this miRNA was generally reduced in pan-gastrointestinal cancers, except for colon adenocarcinoma compared with normal tissues (p < 0.05; Figure 1c-1F), which hinted at possibly crucial functions of miR-30e-5p in cancers, particularly pan-gastrointestinal cancers.

### miR-30e-5p impeded PCa cell proliferation and accelerated apoptosis

**Figure 2.**
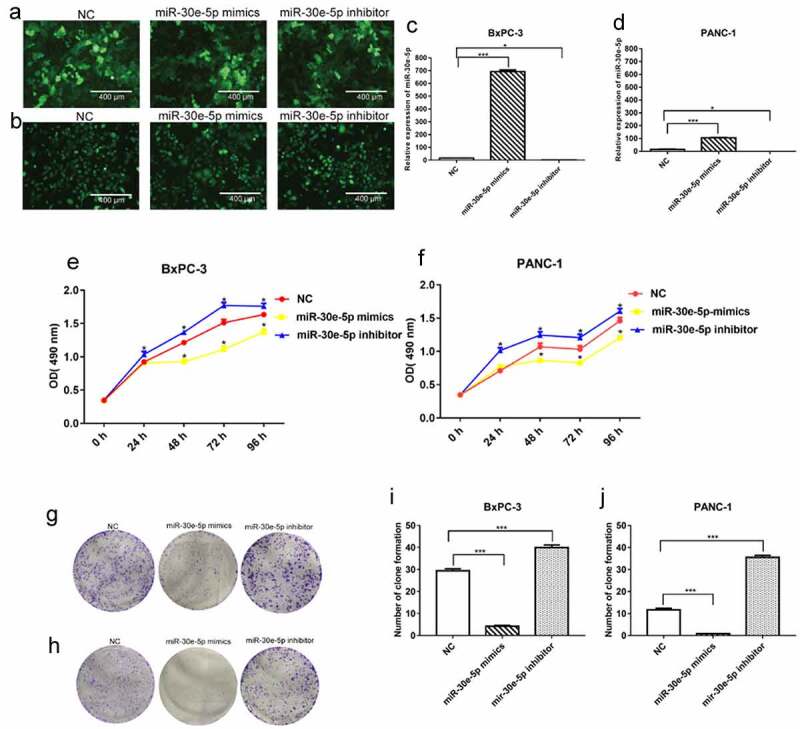
miR-30e-5p could impede PCa cell proliferation. (A, B) Fluorescence expression levels of BxPC-3 and PANC-1 cells transfected with the NC and miR-30e-5p mimics and miR-30e-5p inhibitor in each group. (C, D) miR-30e-5p expression levels of BxPC-3 and PANC-1 cells in each group by qRT-PCR. (E, F) Cell viability of BxPC-3 and PANC-1cells in each group after transfection with miR-30e-5p. (G-J) Number of clone formation of BxPC-3 and PANC-1cells in each group. ***p < 0.001; **p < 0.01; *p < 0.05. Magnification: 100X.

**Figure 3.**
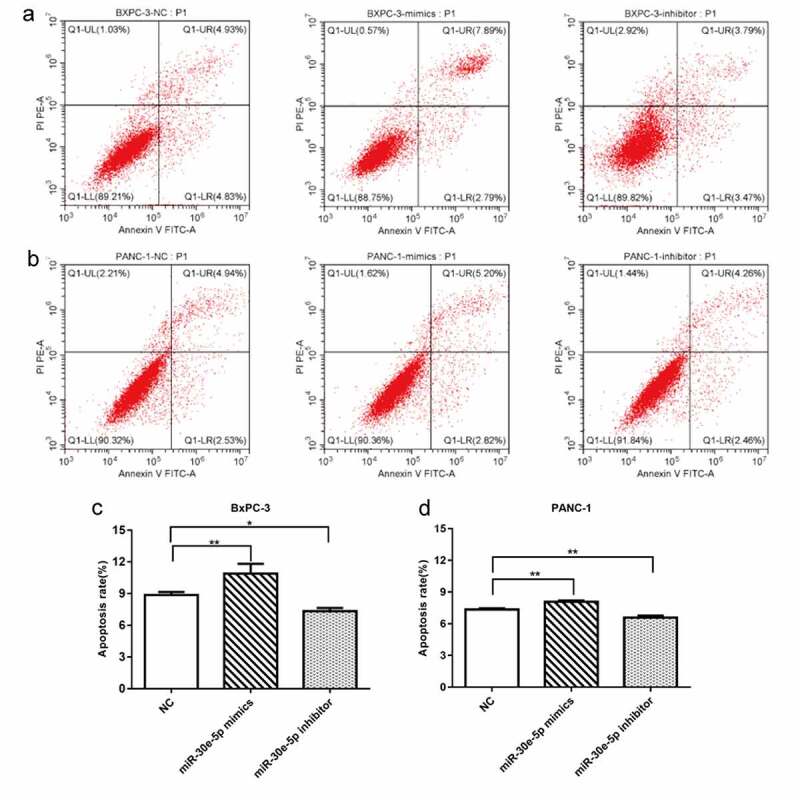
miR-30e-5p could promote PCa cell apoptosis. (A) Flow cytometry of BxPC-3 cells in each group. (B) Flow cytometry of PANC-1 cells in each group. (C) Histogram of apoptosis rate of BxPC-3 cells in each group. (D) Histogram of apoptosis rate of PANC-1 cells in each group. The apoptosis was detected 48 hours after transfection, respectively. ***p < 0.001; **p < 0.01; *p < 0.05.

BxPC-3 and PANC-1 cells are common and easily transfected cells in PCa, which makes it convenient to perform further experiments. Thus, in the present study, we constructed PCa cell lines in which miR-30e-5p was overexpressed or inhibited by transfection of BxPC-3, as well as PANC-1 cells with miR-30e-5p mimics or inhibitors, respectively. Immunofluorescence assays confirmed the transfection of these cell lines (Figure 2a, 2B). In addition, compared with the negative control (NC) group, qRT-PCR exhibited a substantially increased expression of this miRNA in the mimics group and a significantly decreased expression in the inhibitor group (p < 0.05; Figure 2c, 2D). Subsequently, cell proliferation assays and clone formation experiments were performed to evaluate cell proliferation. Compared with the NC group, the miR-30e-5p mimic group markedly blocked BxPC-3 and PANC-1 cell proliferation, while the miR-30e-5p inhibitor group induced the rapid proliferation of these cells (p < 0.05; Figure 2e, 2F). Furthermore, the number of clones was significantly higher in the miR-30e-5p mimic group than in the NC group, and clone formation was remarkably improved in the miR-30e-5p inhibitor group (p < 0.05; Figure 2g–2J). Flow cytometry was used to examine the rate of apoptosis in PCa cell lines. miR-30e-5p mimics group had a significantly apparent increasing apoptosis rate than the NC group, and conversely, apoptosis was suppressed in the miR-30e-5p inhibitor group (p < 0.05; Figure 3).

### miR-30e-5p repressed PCa cells migration and invasion

**Figure 4.**
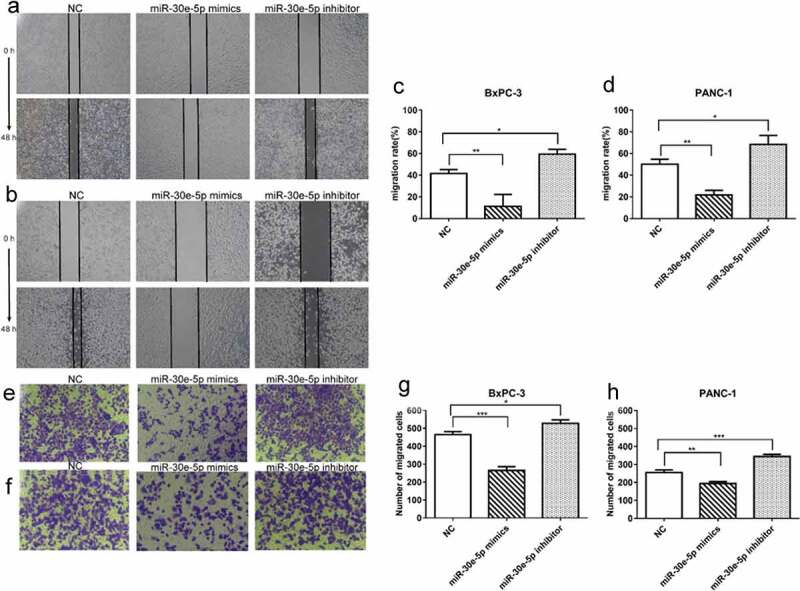
miR-30e-5p could represse PCa cells migration and invasion. (A, B) Micrographs of migration distance of BxPC-3 and PANC-1 cells in each group. (C, D) Histogram of migration ratio of BxPC-3 and PANC-1 cells in each group. The migration distance was detected 48 hours after transfection, respectively. (E, F) Micrographs of the number of BxPC-3 cells and PANC-1 cells that passing through the stromal membrane in each group. (G, H) Histogram of the number of BxPC-3 cells and PANC-1 cells that passing through the stromal membrane in each group. The number of cells crossing the stromal membrane was detected 48 hours after transfection, respectively. ***p < 0.001; **p < 0.01; *p < 0.05. Magnification: 100X.

A wound healing assay demonstrated a reduction in the migration distance of BxPC-3 and PANC-1 cells in the miR-30e-5p mimics group compared with the NC group, whereas the migration distance was significantly increased in the inhibitor group (p < 0.05; Figure 4a-4D). Furthermore, Transwell assays revealed that upregulation of miR-30e-5p expression hindered PCa cell invasion ability, while downregulating its expression enhanced cell invasion (p < 0.05; Figure 4g-4H).

### Influence of miR-30e-5p on the transcriptome of PCa cells

**Figure 5.**
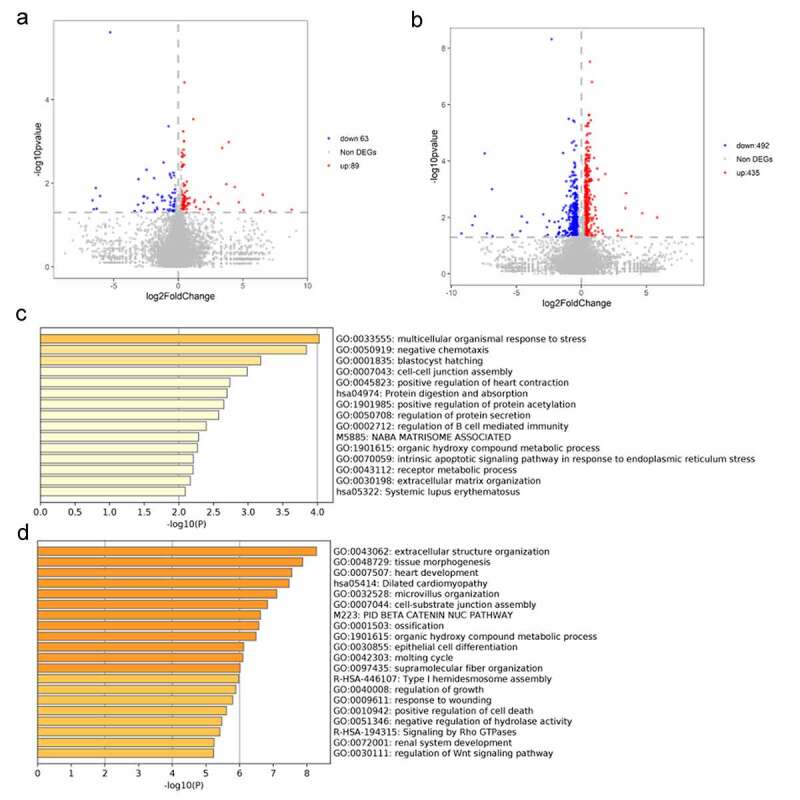
Functional annotation for differential expressed genes. (A) Volcano plot of DEGs from comparison between miR-30e-5p mimics and NC groups. (B) Volcano plot of DEGs from comparison between miR-30e-5p inhibitor and NC groups. (C) GO terms for DEGs in miR-30e-5p mimics group; (D) GO terms for DEGs in miR-30e-5p inhibitor group.

Given the above findings, we aimed to identify the mechanism by which miR-30e-5p affects the development of PCa. For this purpose, RNA was extracted from three groups of PANC-1 cells transfected with miR-30e-5p mimics, miR-30e-5p inhibitors, or empty vectors to perform RNA sequencing. A total of 152 genes were differentially expressed between the miR-30e-5p mimics and NC groups, with 89 upregulated and 63 downregulated genes (Figure 5a). These differentially expressed genes (DEGs) were functionally annotated using Metascape. This analysis showed that these DEGs were mainly concentrated in the following Gene Ontology (GO) terms: cell-cell junction assembly, regulation of B cell-mediated immunity, and extracellular matrix organization (Figure 5c). Similarly, 927 DEGs (including 435 upregulated genes and 492 downregulated) were identified between the miR-30e-5p inhibitor and NC groups (Figure 5b). They were mainly enriched for the following GO terms: epithelial cell differentiation, regulation of growth, response to wounding, and positive regulation of cell death (Figure 5d).

### SNAI1 was identified as a target gene of miR-30e-5p

**Figure 6.**
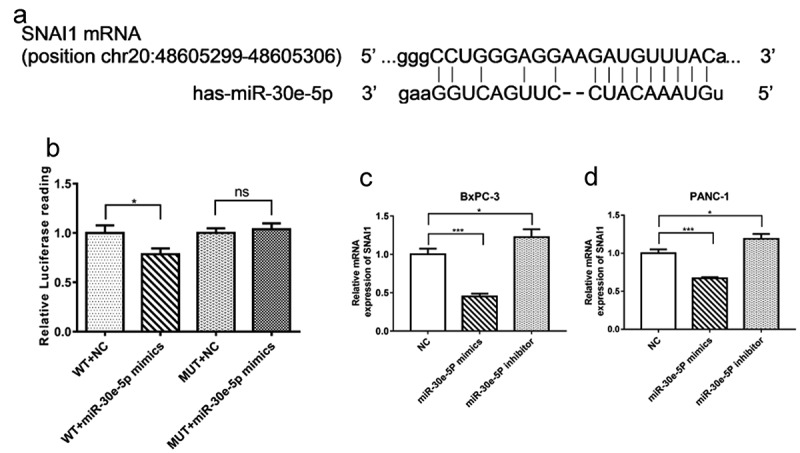
SNAI1 was identified as a target gene of miR-30e-5p in PCa. (A) Bioinformatic analysis for predicting binding sites of miR-30e-5p in SNAI1 by Targetscan website. (B) Luciferase assay for luciferase reporters with wild-type or mutant SNAI1 3’ UTR relative to Renilla luciferase activity in 293T cells transiently transfected with the negative control and miR-30e-5p mimic. (C, D) The SNAI1 expression levels of BxPC-3 and PANC-1 cells in each group. ***p < 0.001; **p < 0.01; *p < 0.05. ns: no significance.

To further probe the molecular mechanism of miR-30e-5p in PCa, StarBase 3.0 was utilized to identify the putative target genes. As shown in Figure 6a, SNAI1 was predicted to associate with this miRNA based on PITA, DIANA-microT, miRanda, and TargetScan analyses. Then to test whether SNAI1 was directly targeted by miR-30e-5p, we performed dual-luciferase reporter assays. Compared with the NC group, the luciferase activity of wild-type SNAI1 3-UTR was limited in the miR-30e-5p mimics group (p < 0.05, Figure 6b). There was no difference in the luciferase activity of the mutant SNAI1 3’-UTR between the miR-30e-5p mimics and NC groups (Figure 6b). In addition, an obvious suppression caused by miR-30e-5p overexpression was observed in the mRNA expression level of SNAI1 in BxPC-3 and PANC-1 cells. Conversely, SNAI1 expression was enhanced in the miR-30e-5p inhibitor group (p < 0.05, Figure 6c and 6D).

### SNAI1 participated in miR-30e-5p-mediated cell proliferation, apoptosis and migration in PCa

**Figure 7.**
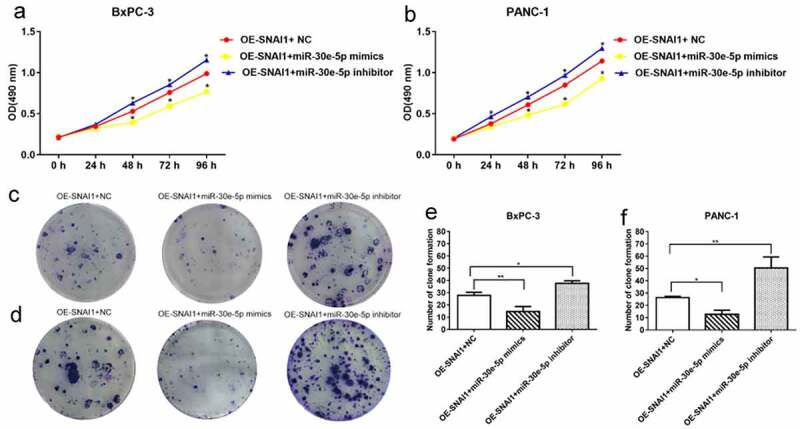
SNAI1 participated in miR-30e-5p-mediated cell proliferation. (A, B) Cell viability of BxPC-3 and PANC-1cells in each group after infected with OE-SNAI1 lentivirus and co-transfected with miR-30e-5p. (C-F) Number of clone formation of BxPC-3 and PANC-1cells in each group after infected with OE-SNAI1 lentivirus and co-transfected with miR-30e-5p. ***p < 0.001; **p < 0.01; *p < 0.05. Magnification: 100X. Abbreviations: OE: Over Expressed.

**Figure 8.**
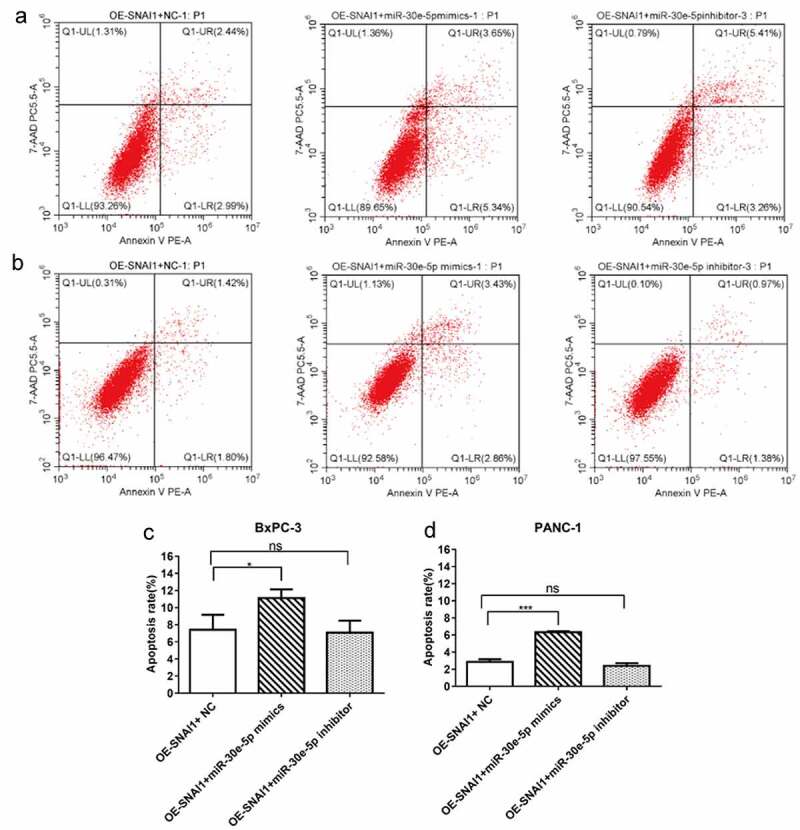
SNAI1 participated in miR-30e-5p-mediated cell apoptosis. (A, B) Flow cytometry of BxPC-3 and PANC-1 cells in each group. (C, D) Histogram of apoptosis rate of BxPC-3 cells and PANC-1 in each group. The apoptosis was detected 48 hours after co-transfection, respectively. ***p < 0.001; **p < 0.01; *p < 0.05.

**Figure 9.**
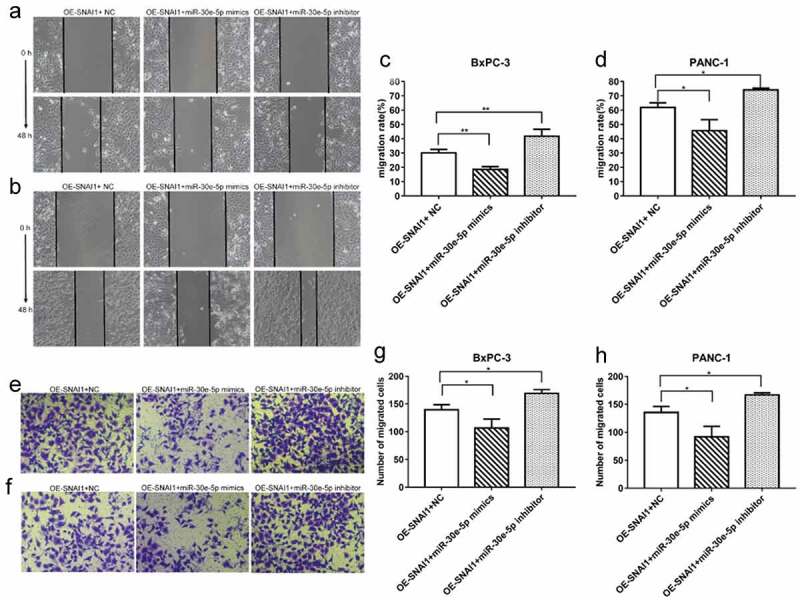
SNAI1 participated in miR-30e-5p-mediated cell migration and invasion. (A, B) Micrographs of migration distance of BxPC-3 and PANC-1 cells in each group. (C, D) Histogram of migration ratio of BxPC-3 and PANC-1 cells in each group. The migration distance was detected 48 hours after co-transfection, respectively. (E, F) Micrographs of the number of BxPC-3 and PANC-1 cells that passing through the stromal membrane in each group. (G, H) Histogram of the number of BxPC-3 and PANC-1 cells that passing through the stromal membrane in each group. The number of cells crossing the stromal membrane was detected 48 hours after co-transfection, respectively. Magnification: 100X.

PCa (BxPC-3 and PANC-1) cells of the miR-30e-5p mimics, inhibitors, and empty vector groups were transfected with a vector overexpressing SNAI1 (OE-SNAI1). In contrast to the OE-SNAI1+ NC group, the OE-SNAI1+ miR-30e-5p mimics group obviously inhibited cell growth, while in contrast, the OE-SNAI1+ miR-30e-5p inhibitor boosted cell proliferation (p < 0.05; Figure 7a-7F). Flow cytometry analysis showed that the OE-SNAI1+ miR-30e-5p mimic group had an increased apoptosis rate compared with the OE-SNAI1+ NC group (p < 0.05, Figure 8). However, there was no obvious change between the OE-SNAI1+ NC and OE-SNAI1+ miR-30e-5p inhibitor groups. Additionally, the number of invading cells was reduced and the migratory distance was shortened in the OE-SNAI1+ miR-30e-5p mimic group compared with the OE-SNAI1+ NC group (p < 0.05, Figure 9). In contrast, when PCa cells were transfected with miR-30e-5p inhibitor and OE-SNAI1, invasion and migration were markedly increased (p < 0.05, Figure 9).

### miR-30e-5p-induced EMT phenotypes

**Figure 10.**
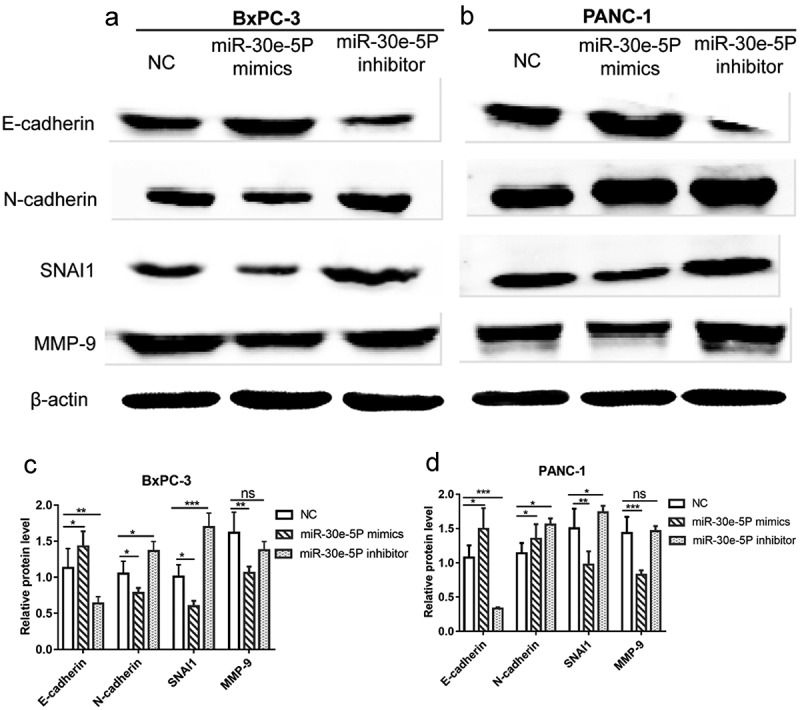
miR-30e-5p could influence the expressions of SNAI-mediated EMT pathway related proteins in PCa. (A, B) Image of western blotting of target proteins (E-cadherin, N-cadherin, SNAI1 and MMP-9) in BxPC-3 and PANC-1 cells in each group after transfection with miR-30e-5p; (C, D) Histogram of relative protein expression levels of target proteins in BxPC-3 and PANC-1 cells in each group. ***p < 0.001; **p < 0.01; *p < 0.05. ns: no significance. EMT: epithelial-mesenchymal transition.

**Figure 11.**
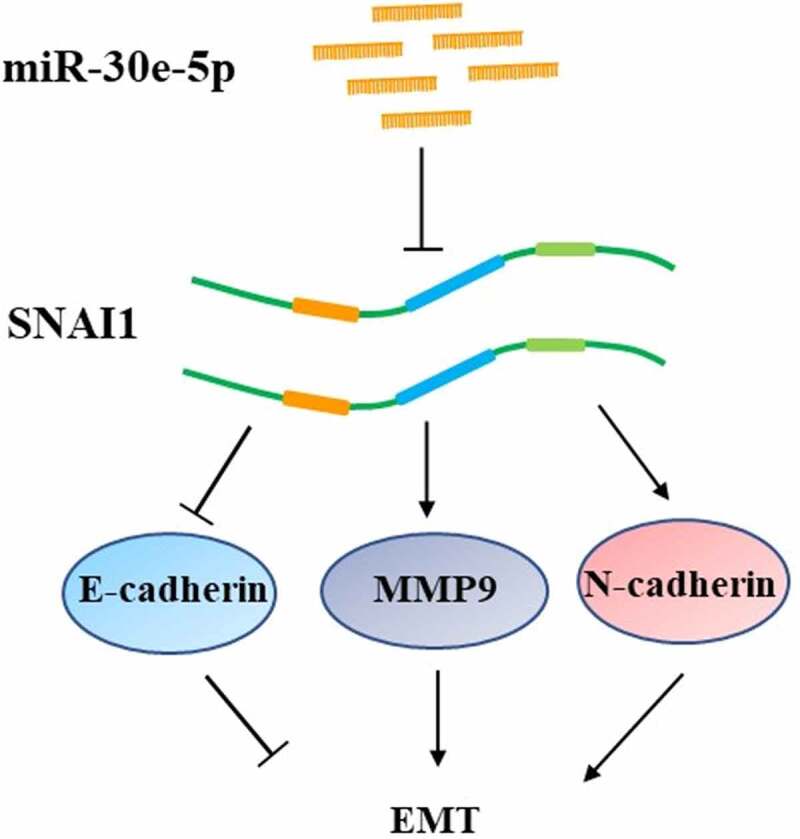
A schematic model of miR-30e-5p targeting SNAI1 to regulate EMT on pancreatic cancer.

In addition to invasion and migration, EMT is a pivotal process in cancer metastasis. Hence, to clarify the role of miR-30e-5p in EMT progression in PCa and the role of SNAI1 in miR-30e-5p-induced EMT phenotypes, BxPC-3 and PANC-1 cells were transfected with miR-30e-5p inhibitor, miR-30e-5p mimics, or NC. Western blot analysis showed that, in contrast to the NC group, E-cadherin was expressed at a higher level in the miR-30e-5p mimics group, whereas it was expressed at a lower level in the miR-30e-5p inhibitor group (p < 0.05; Figure 10). In addition, the expression of N-cadherin, SNAI1, and MMP-9 was examined. Compared with the NC group, N-cadherin expression was obviously decreased in the miR-30e-5p mimic group in BxPC-3 cells, but it was not downregulated in the miR-30e-5p mimic group in PANC-1 cells. When miR-30e-5p was inhibited, N-cadherin expression was significantly elevated in the two PCa cells. Additionally, the expression of SNAI1 was significantly diminished in the miR-30e-5p mimics group compared with the NC group, while their expression levels were increased in the miR-30e-5p inhibitor group (p < 0.05; Figure 10). Similarly, the expressions of MMP-9 in the two PCa cells were both evidently reduced after overexpressing miR-30e-5p, but there is no change of MMP-9 expression after inhibiting miR-30e-5p (Figure 10). These results illustrated that miR-30e-5p plays a vital role in the SNAI-mediated EMT process in PCa (Figure 11).

## Discussion

Pancreatic cancer (PCa) is a highly malignant tumor characterized by rapid progression and poor prognosis of patients. Although its diagnosis and treatment have improved, the 5-year overall survival rate remains only 9% in the United States [3]. MiRNAs may act as biomarkers for cancer diagnosis, risk stratification, and outcome prediction, and may be used as therapeutic targets [32,33]. In this study, we found that miR-30e-5p functions as a tumor inhibitor in the regulation of proliferation, apoptosis, migration, and EMT of PCa cells by targeting SNAI1, suggesting that miR-30e-5p and SNAI1 play crucial roles in PCa.

It is well established that miR-30e-5p inhibits the metastasis of squamous cell carcinoma of the head and neck (SCCHN) by affecting EMT and impeding SCCHN angiogenesis both in vitro and in vivo [34]. Astrocyte elevated gene-1 protein was identified as a target gene of miR-30e-5p, and partly participates in miR-30e-5p-mediated metastasis and angiogenesis [34]. Alike, it was revealed that miR-30e-5p depletes the occurrence of non-small cell lung cancer by downregulating ubiquitin-specific peptidase 22 and affecting the sirtuin 1/Janus kinase/signal transducer and activator of transcription 3 signaling pathway [35]. Another study indicated that p53-induced miR-30e-5p resists colorectal carcinoma invasion and metastasis by targeting the integrin subunits, beta 1 and alpha 6 [11]. Recent study also showed that miR-30e-5p is a hub miRNA that may be used as an effective biomarker and a novel therapeutic target in osteosarcoma [36]. However, the specific relationship between miR-30e-5p and PCa has not yet been reported. In this study, miR-30e-5p expression levels were found to be downregulated in PCa tissues compared to that in the adjacent normal tissues. The same result was observed in other pan-gastrointestinal cancerous tissues, except in colon cancer. These results emphasize the importance of miR-30e-5p in carcinogenesis. Subsequently, by utilizing gain- and loss-of miR-30e-5p experiments in vitro, we ascertained that this miRNA may repress cell growth, invasion, and migration, as well as promote cell apoptosis in PCa cell lines. Furthermore, we found that the apoptosis rate of BxPC-3 cells was higher than that of PANC-1 cells. Universally known, BxPC-3 cells represent an early stage of PCa, whereas PANC-1 cells represent the advanced stage [37]. Dhanya Nambiar et al. reported that silibinin induces a strong dose-dependent G1 cell cycle arrest in BxPC-3 cells, while only causing a middling response in PANC-1 cells. Moreover, the percentage of apoptotic cell population was increased up to threefold in BxPC-3 cells after treatment with silibinin, but only trifling changes were observed in PANC-1 cells. Silibinin shows differential anticancer efficacy against BxPC-3 and PANC-1 cells [37]. Interestingly, RNA sequencing results further confirmed that miR-30e-5p plays an important role in the regulation of growth, cell death, and epithelial cell differentiation.

Next, we validated SNAI1 as a direct target gene of miR-30e-5p in PCa using bioinformatics analysis and experimental methods. We also found that SNAI1 abates the expression of E-cadherin by binding to gene promoters related to cell adhesion and participates in the initiation of EMT [38,39]. Recently, Dong et al. reported that DNA topoisomerase II alpha can trigger EMT and accelerate the progression of hepatocellular carcinoma by monitoring Snail expression [40]. Previous studies have reported abnormal expression of SNAI1 in various malignant tumors. For example, in PCa mouse models with *KRAS* mutations, SNAI1 promotes fibrosis by elevating the transforming growth factor-β signaling transduction, which further accelerates PCa development [41]. Mikami et al. and Cai provided evidence that Snail1 may serve as a potential prognostic biomarker and therapeutic target by mediating EMT in clear cell renal cell carcinoma [42,43]. Additionally, Deep et al. described the key role of SNAI1 in the aggressiveness of prostate cancer cells by alleviating E-cadherin expression [39]. Hence, in the present study, we not only defined SNAI1 as a target gene of miR-30e-5p, but also performed a series of experiments to explain the relationships among miR-30e-5p, SNAI1, and EMT. Through co-transfection of PCa cell lines with SNAI1 and miR-30e-5p-inhibitors, miR-30e-5p-mimics, or empty vectors, cell proliferation and migration were found to be enhanced, while cell apoptosis was retarded in the OE-SNAI1 + miR-30e-5p inhibitor group. In contrast, there was a marked decline in cell growth and migration, and an increase in apoptosis in the OE-SNA11 + miR-30e-5p mimic group. Gain- and loss-of miR-30e-5p in in vitro experiments revealed that miR-30e-5p enhanced the protein expression of E-cadherin, while ameliorating the expression levels of N-cadherin, MMP-9, and SNAI1. EMT is the pivotal molecular mechanism involved in cancer metastasis, and E-cadherin is the characteristic marker of EMT; however, other markers, including N-cadherin, MMP-9, and SNAI1 are also important for EMT [44]. These results indicate that miR-30e-5p acts as an inhibitor of PCa carcinogenesis and progression by targeting SNAI1 and mediating EMT, suggesting that miR-30e-5p and SNAI1 may be used as novel therapeutic targets for PCa treatment.

This study has some limitations that can be addressed in future studies. First, more clinical samples should be collected to examine the expression levels of miR-30e-5p and SNAI1 as well as their association with various clinical parameters, such as tumor grade and stage. Second, the findings of this study can be strengthened by performing in vivo experiments. Despite these limitations, this is the first study to explore the role of miR-30e-5p in PCa using clinical tissue samples and performing in vitro experiments. These results provide novel insights into the molecular mechanisms of miR-30e-5p in PCa.

## Conclusion

In conclusion, this study reveals the underlying mechanism by which miR-30e-5p mitigates PCa cell proliferation and migration by restraining SNAI-mediated EMT. Therefore, targeting miR-30e-5p and SNAI1 may be used as a novel therapeutic strategy for PCa treatment.

## Supplementary Material

Supplemental Material
