# Supplementary material for: The effect and mechanism of miR-30e-5p targeting SNAI1 to regulate epithelial-mesenchymal transition on pancreatic cancer
Source: Bioengineered. 2022 Mar 18;13(4):8013–28. doi: 10.1080/21655979.2022.2050880 (PMC9161848; doi:10.1080/21655979.2022.2050880)

**Raw data of microscopy images in figure 2**

BxPC-3 inhibitor 100X


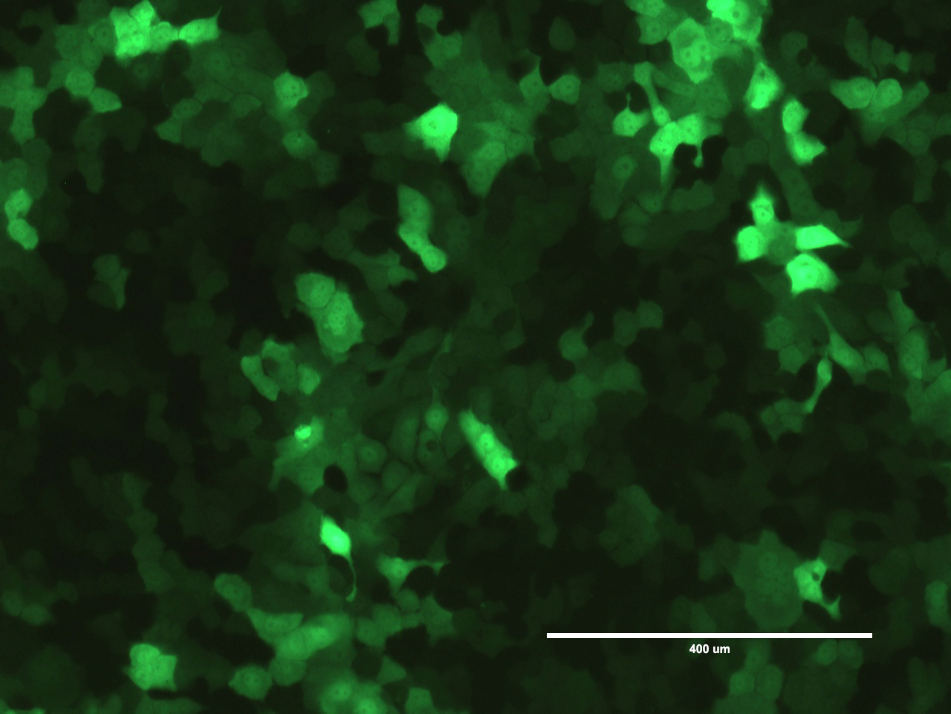


BxPC-3 mimic 100X


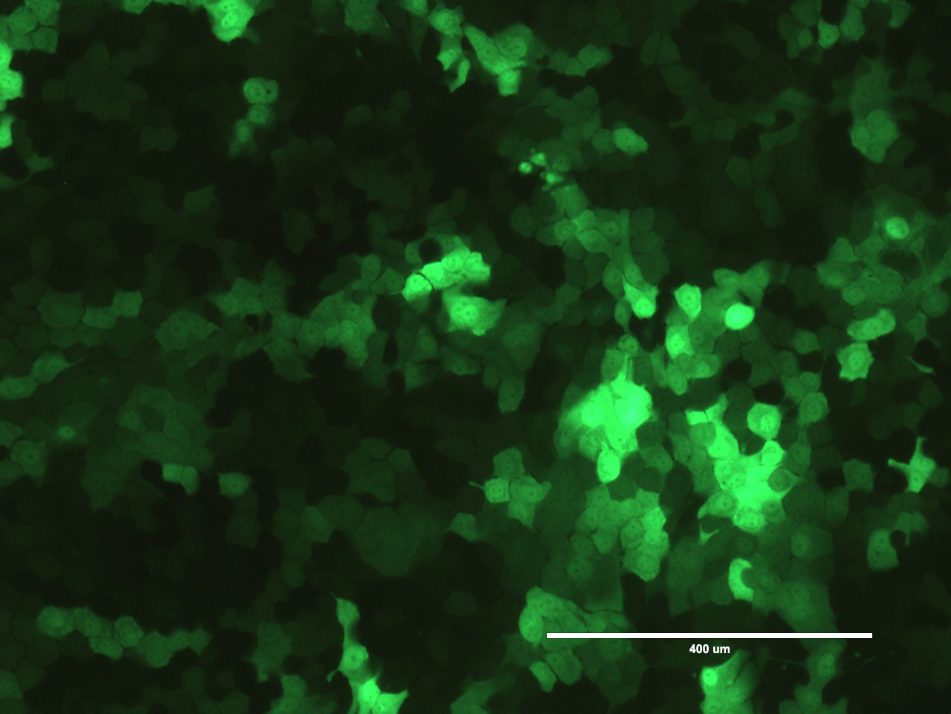


BxPC-3 NC 100X


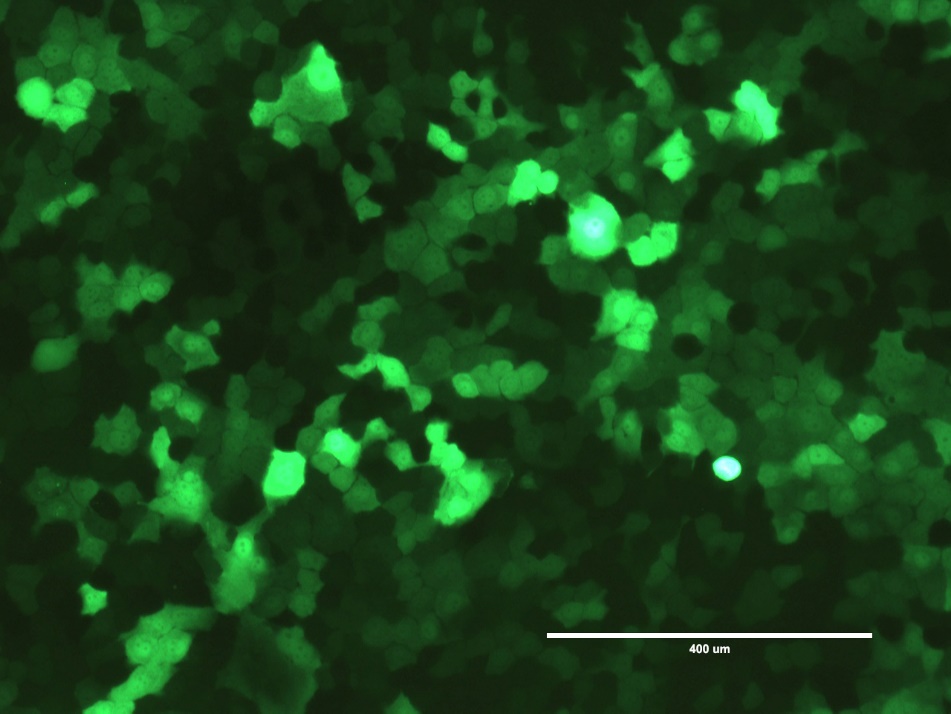


PANC-1 inhibitor 100X


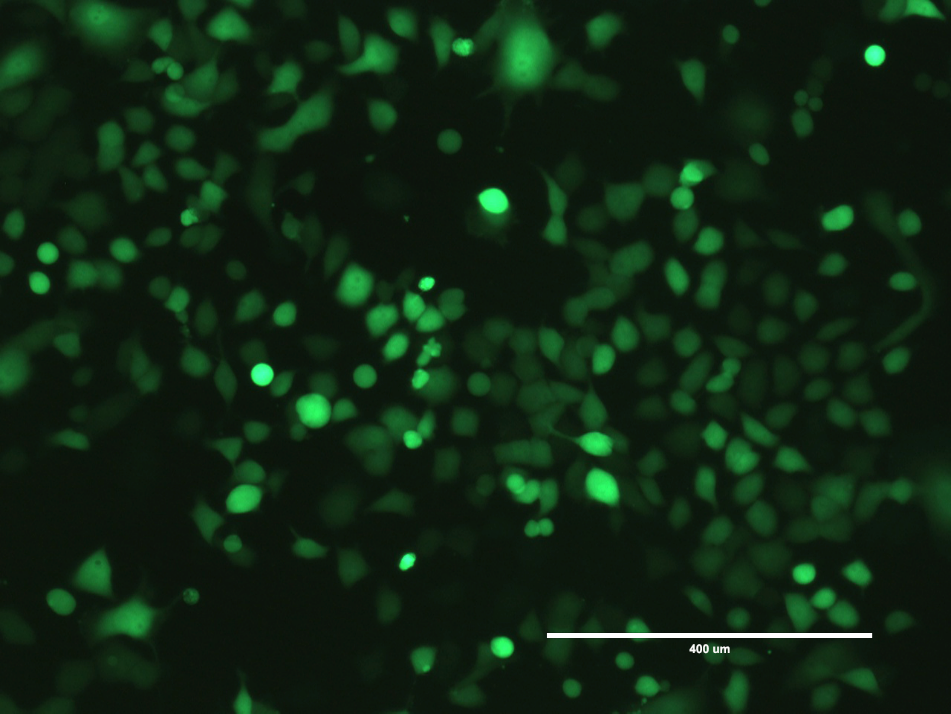


PANC-1 mimic 100X


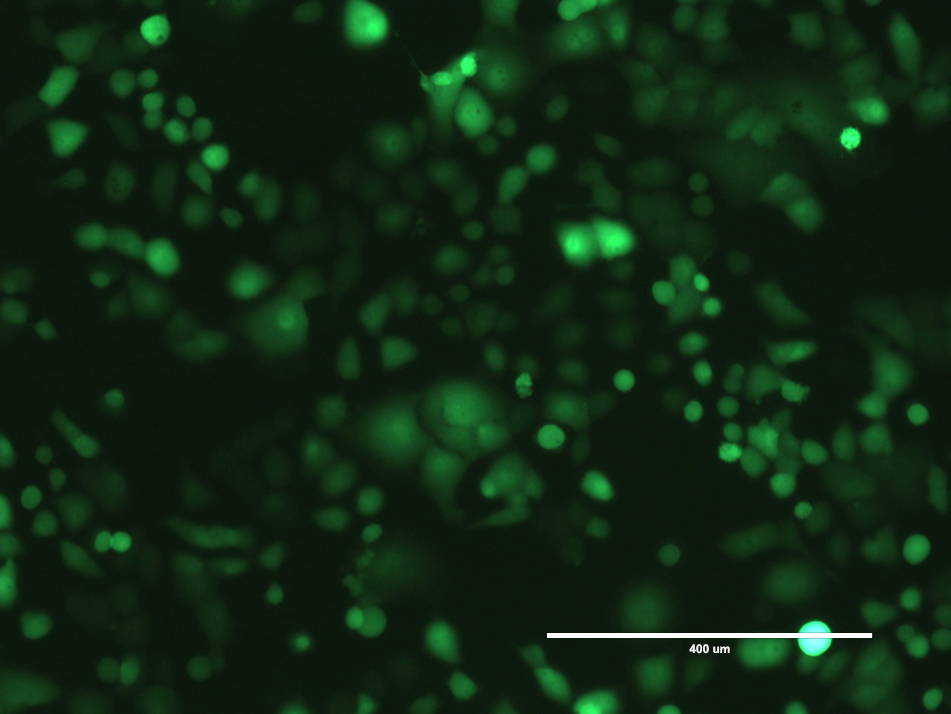


PANC-1 NC 100X


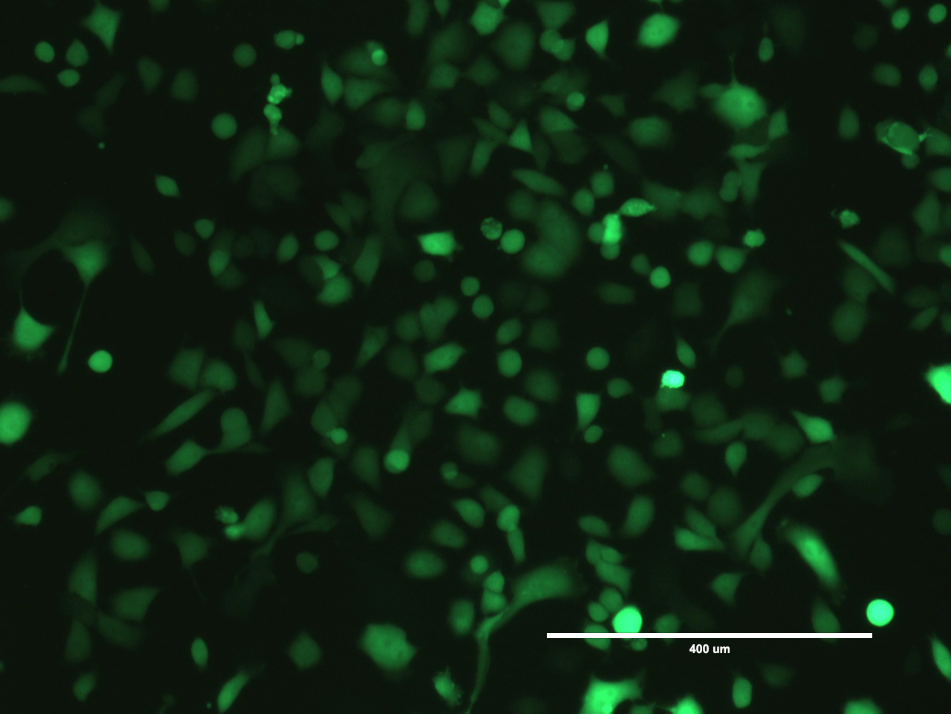

Supplement: Supplemental Material [file KBIE_A_2050880_SM5944.zip › supplementary/Raw data microscopy images figure 2.docx]
