# Supplementary material for: The effect and mechanism of miR-30e-5p targeting SNAI1 to regulate epithelial-mesenchymal transition on pancreatic cancer
Source: Bioengineered. 2022 Mar 18;13(4):8013–28. doi: 10.1080/21655979.2022.2050880 (PMC9161848; doi:10.1080/21655979.2022.2050880)

**Raw data of western blots in figure 10**

BxPC-3 E-cadherin











BxPC-3 MMP-9











BxPC-3 N-cadherin











BxPC-3 SNAI1











BxPC-3 β-actin


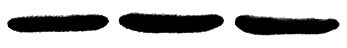


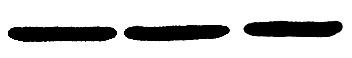


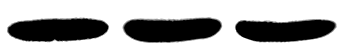


PANC-1 E-cadherin











PANC-1 MMP-9











PANC-1 N-cadherin











PANC-1 SNAI1


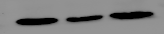








PANC-1 β-actin


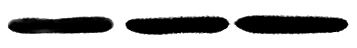


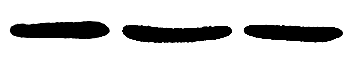


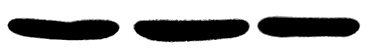

Supplement: Supplemental Material [file KBIE_A_2050880_SM5944.zip › supplementary/Raw data western blots figure 10.docx]
