# Supplementary material for: The effect and mechanism of miR-30e-5p targeting SNAI1 to regulate epithelial-mesenchymal transition on pancreatic cancer
Source: Bioengineered. 2022 Mar 18;13(4):8013–28. doi: 10.1080/21655979.2022.2050880 (PMC9161848; doi:10.1080/21655979.2022.2050880)
Supplement: Supplemental Material [file KBIE_A_2050880_SM5944.zip › supplementary/suppl.pdf]

广西医科大学第一附属医院
THE FIRST AFFILIATED HOSPITAL OF
GUANGXI MEDICAL UNIVERSITY

FIRST AFFILIATED HOSPITAL of GUANGXI MEDICAL UNIVERSITY
ETHICAL REVIEW COMMITTEE
Approval Notice

Approval Number: 2021(KY-226)

Title: Regulation of invasion and metastasis in pancreatic cancer by IFN-γ/MACC1/MMP1 axis

Research Contents: Pancreatic cancer is one of the most common malignant tumors of digestive tract in China, and ranks seventh in the global cancer mortality rate, with a 5-year survival rate of only 8%. In China, the incidence and mortality of pancreatic cancer are in the top 10 malignant tumors. Pancreatic cancer has a poor prognosis and a high mortality rate, which is related to the high degree of malignancy of most pancreatic cancers, often at an advanced stage at the time of diagnosis, and loss of surgical treatment opportunities. These characteristics of pancreatic cancer are related to the strong invasion and metastasis ability and lack of pancreatic cancer cells. Effective treatment is closely related. Therefore, it is very important to study the mechanism of invasion and metastasis of pancreatic cancer. This project intends to explore and verify the role and molecular mechanism of the oncogene MACC1, which is closely related to the invasion and metastasis of pancreatic cancer, in the invasion and metastasis of pancreatic cancer based on the preliminary experiments, combined with human pancreatic cancer tissue specimens, cell models and animal experiments. Provide a new scientific basis for the invasion and metastasis of pancreatic cancer.

Applicant: Qin Shanyu

Application Department:the National Natural Science Foundation of China

Acknowledgement: This study was supported by supported by grants from

Date of Application: October 11, 2021

Date of Approval: October 12, 2021

Conclusion: This project fully considered and protected the rights and interests of the study objects. It meets the criteria of Ethical Review Committee. The Medical Ethics Committee of First Affiliated Hospital of Guangxi Medical University has approved the protocol.

Signature: Songqing He

Songqing He

(Vice) Director of Ethical Review Committee

First Affiliated Hospital of Guangxi Medical University

Date: Oct 12, 2021
