# Supplementary figures and images for: The effect and mechanism of miR-30e-5p targeting SNAI1 to regulate epithelial-mesenchymal transition on pancreatic cancer
Source: Bioengineered. 2022 Mar 18;13(4):8013–28. doi: 10.1080/21655979.2022.2050880 (PMC9161848; doi:10.1080/21655979.2022.2050880)

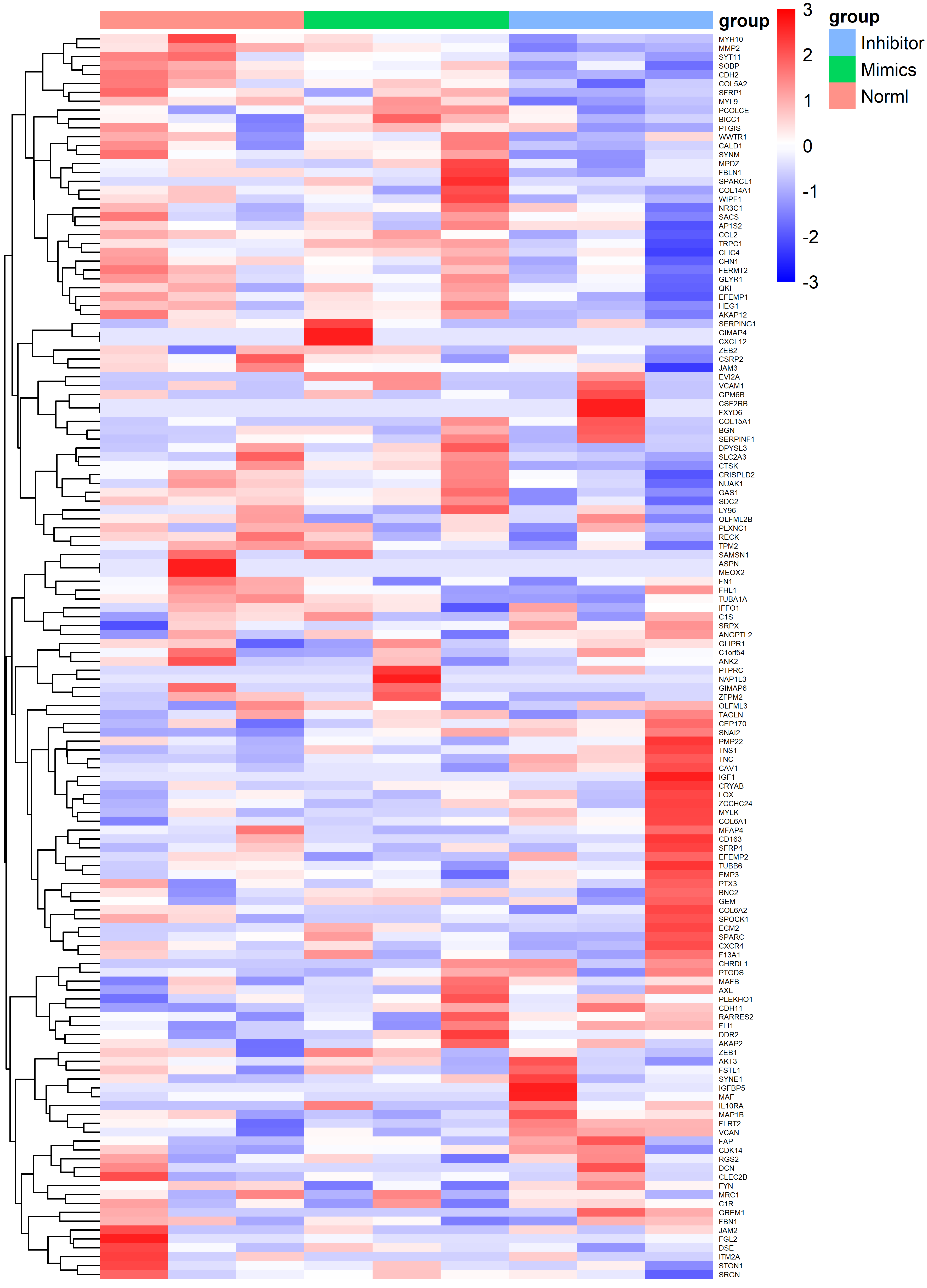

Supplement: Supplemental Material [file KBIE_A_2050880_SM5944.zip › supplementary/Figure S1 A heatmap for the EMT UP signature.tiff]

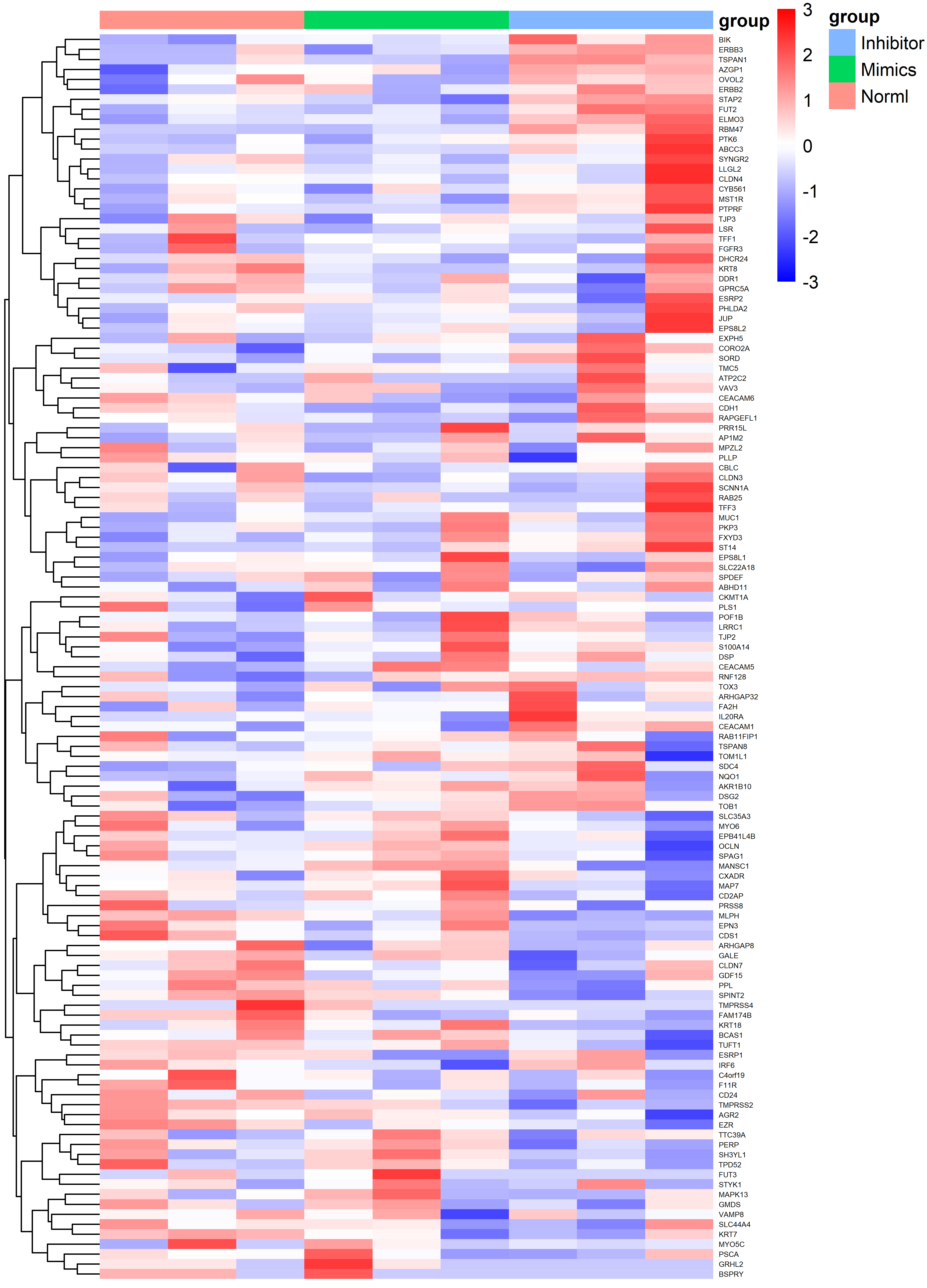

Supplement: Supplemental Material [file KBIE_A_2050880_SM5944.zip › supplementary/Figure S2 A heatmap for the EMT DOWN signature.tiff]
